# Analysis of the cytoskeleton organization and its possible functions in male earthworm germ-line cysts equipped with a cytophore

**DOI:** 10.1007/s00441-016-2398-6

**Published:** 2016-04-12

**Authors:** Karol Małota, Piotr Świątek

**Affiliations:** Department of Animal Histology and Embryology, University of Silesia, Bankowa 9, 40-007 Katowice, Poland

**Keywords:** Intercellular bridges, F-actin, Microtubules, Spermatogenesis, Cytoskeletal drugs

## Abstract

We studied the organization of F-actin and the microtubular cytoskeleton in male germ-line cysts in the seminal vesicles of the earthworm *Dendrobaena veneta* using light, fluorescent and electron microscopy along with both chemically fixed tissue and life cell imaging. Additionally, in order to follow the functioning of the cytoskeleton, we incubated the cysts in colchicine, nocodazole, cytochalasin D and latrunculin A. The male germ-line cells of *D. veneta* are interconnected via stable intercellular bridges (IB), and form syncytial cysts. Each germ cell has only one IB that connects it to the anuclear central cytoplasmic mass, the cytophore. During the studies, we analyzed the cytoskeleton in spermatogonial, spermatocytic and spermatid cysts. F-actin was detected in the cortical cytoplasm and forms distinct rings in the IBs. The arrangement of the microtubules changed dynamically during spermatogenesis. The microtubules are distributed evenly in whole spermatogonial and spermatocytic cysts; however, they primarily accumulate within the IBs in spermatogonia. In early spermatids, microtubules pass through the IBs and are present in whole cysts. During spermatid elongation, the microtubules form a manchette while they are absent in the cytophore and in the IBs. Use of cytoskeletal drugs did not alter the general morphology of the cysts. Detectable effects—the occurrence of nuclei in the late spermatids and manchette fragments in the cytophore—were observed only after incubation in nocodazole. Our results suggest that the microtubules are responsible for cytoplasmic/organelle transfer between the germ cells and the cytophore during spermatogenesis and for the positioning of the spermatid nuclei.

**Electronic supplementary material:**

The online version of this article (doi:10.1007/s00441-016-2398-6) contains supplementary material, which is available to authorized users.

## Introduction

The formation of the syncytial groups of cells, which are usually called cysts, clusters, nests or isogenic groups, seems to be a conservative and widespread phase of animal gametogenesis (Pepling et al. 1999; Ventelä 2006; Haglund et al. 2011; Greenbaum et al. 2011). Within germ-line cysts, there is a cytoplasmic continuity between interconnected cells due to the presence of broad (up to 10 μm; Ventelä 2006; Haglund et al. 2011) cytoplasmic channels (stable intercellular bridges, cytoplasmic bridges, ring canals) that allow the cytoplasm to be shared. The intercellular bridges (IBs) are modified contractile rings that do not close during late cytokinesis (there is no abscission) and, as a result, the cytoplasm of sister cells is common (Greenbaum et al. 2011; Haglund et al. 2011). It has been experimentally demonstrated that in model species such as *Caenorhabditis elegans*, *Drosophila melanogaster* and *Mus musculus* the absence or severe disorders in the formation and functioning of the IBs are connected with infertility (e.g., Robinson et al. 1994; Brill et al. 2000; Maddox et al. 2005; Greenbaum et al. 2006; Green et al. 2011; Yamamoto et al. 2013; Lorès et al. 2014). It is widely accepted that the interconnections of germ cells into syncytial clusters regulate and synchronize germ cell development (Pepling et al. 1999; Guo and Zheng 2004; Ventelä 2006; Greenbaum et al. 2011; Haglund et al. 2011; Amini et al. 2014). However, it seems that the actual role of a cyst is sex-dependent. In males, cysts are formed similarly in both invertebrate and vertebrate animals (Roosen-Runge 1977; Adiyodi and Adiyodi 1983). Male germ cells develop and differentiate synchronously within the cysts and the gene products and even organelles may be shared between cells and therefore haploid spermatids remain phenotypically diploid in late spermatogenesis (Braun et al. 1989; Morales et al. 2002; Ventelä et al. 2003). On the other hand, in female gametogenesis, matters are more complicated. Germ-line cysts are completely absent in some taxa (e.g., Büning 1994; Tworzydło et al. 2014), whereas in vertebrate species such as *Xenopus laevis* or *M. musculus* the female cysts are transient and germ cells quickly spilt into individual cells and the role of cell clustering is not clear (Pepling and Spradling 1998; Pepling et al. 1999; Kloc et al. 2004, 2008; Greenbaum et al. 2009). Finally, in some invertebrate taxa including insects, only several (sometimes one) cells in a cyst pass meiosis, gather nutrients and become oocytes and the rest of the interconnected cells supply the growing oocytes with macromolecules and cell organelles—these cells die late in oogenesis and serve as auxiliary (nurse) cells (Telfer 1975; Büning 1994; Matova and Cooley 2001).

Numerous analyses of developing male and female germ cells have shown that the spatial organization (architecture) of germ-line cysts varies among taxa. Taking only male germ cysts into account, it can be assumed that three types of cysts are predominant: linear, branched and cysts that have a central cytoplasmic mass. In linear clusters, the cells form chains – each germ cell has two IBs that connect them to their neighbors and only the terminal cells have one IB. Linear chains, which are composed of hundreds or even thousands of male germ cells, are widespread in vertebrates (Fawcett et al. 1959; Roosen-Runge 1977; Greenbaum et al. 2011). Some germ cells in branched cysts may have more than two IBs and, as a result, the “branchings” occur in these sites. The branched cysts of male germ cells are best known from some insects, especially *D. melanogaster* (Hime et al. 1996; Eikenes et al. 2013). In some invertebrates, e.g., nematodes, flat worms and annelids, germ cells are not directly interconnected to one another but rather, as a rule, each germ cell in a cyst has only one IB joining it to a common and anuclear cytoplasmic mass (the cytoplasmic core). In the gonads of some nematodes such as *C. elegans*, the cytoplasmic core has the form of a cylinder and is known as the rachis or gonad core (Hirsh et al. 1976; Wolf et al. 1978; L’Hernault 2009). In flat worms and annelids, the cytoplasmic core is traditionally called a cytophore and its shape depends on the developmental stage of the cyst (Davis and Roberts 1983; Olive 1983; Ferraguti 1983; Świątek et al. 2009).

The formation, organization and functioning of male germ-line cysts is best known in the case of branched cysts. Numerous studies, mainly on *D. melanogaster*, have revealed many sophisticated molecular aspects of the biology of the cysts, e.g., the germ-line-specific cytoskeletal-rich cytoplasm called fusome is engaged in the correct segregation of IBs during the subsequent divisions of daughter cells (Hime et al. 1996; Eikenes et al. 2013). Fewer studies have been devoted to germ-line cysts that have a cytoplasmic core. Although we have a powerful model for such studies, i.e., *C. elegans*, the numerous aspects of cyst biology such as the mechanism that governs the formation or the organization of these cysts and the role of the cytoskeleton are far from elucidation. Recently, it has been experimentally shown that the actomyosin cytoskeleton plays an active role in cytoplasmic streaming during oogenesis in *C. elegans* and that *C. elegans* homologs of the protein anillin regulate the stability of IBs (Wolke et al. 2007; Amini et al. 2014). Moreover, morphological studies on clitellate annelids have revealed that the formation of germ-line cysts with a cytophore differs markedly from the one that is known from branched and linear cysts (Świątek et al. 2009). In the latter cases, the IBs are always formed de novo as modified and stabilized contractile rings (Robinson et al. 1994; Robinson and Cooley 1996; Ong and Tan 2010; Greenbaum et al. 2011; Haglund et al. 2011). In clitellate annelids, the specific orientation of mitotic spindles causes the contractile ring of dividing germ cells to merge with a pre-existing bridge and splits it into two new bridges (Świątek et al. 2009).

Clitellate annelids (Clitellata) seem to be a very attractive model for analyses of germ-line cysts that have cells that are clustered around the cytophore. The germ-line clusters that have a cytophore have been found in all the species of Clitellata that have been studied to date, in both male and female gametogenesis (Ferraguti 2000; Jamieson 2006; Świątek et al. 2009; Urbisz et al. 2015). Male germ-line cysts are exceptionally easy to obtain and manipulate. Although the first divisions of spermatogonia occur in the testis, which are usually tiny structures that are not easy to handle, the later spermatogonial divisions, meiosis and spermiogenesis take place in the specialized diverticula of the septa, the so-called seminal vesicles (Jamieson 1992). The seminal vesicles are easy to manipulate and, what is more important, there are hundreds or even thousands of germ cysts freely floating in the coelomic fluid in each seminal vesicle. Moreover, the clustered germ-line cells that are floating within the seminal vesicles are not associated with any somatic cells (Jamieson 1981, 1992), which greatly facilitates their analysis. The different aspects of spermatogenesis (especially spermiogenesis) and sperm structure in Clitellata are well described at the ultrastructural level in many taxa (see Ferraguti 1983, 2000; Jamieson 1981, 1992, 2006 for a review), because the characters that are connected with sperm structure have been widely used in phylogenetic assessments (e.g., Marotta et al. 2008; Marotta and Ferraguti 2009). To date, ultrastructural studies on spermatogenesis and cyst structure in Clitellata have concentrated only on such aspects as the formation of the IBs and cytophore (Martinucci et al. 1977; Świątek et al. 2009), nuclear fragmentation and the multiplication of basal bodies during paraspermiogenesis (Boi et al. 2001; Ferraguti et al. 2002) or the distribution of cytoskeletal elements such as actin- and gelsolin-related proteins (Krüger et al. 2008). We decided to perform systematic studies on the development and functioning of male germ-line cysts in the commercially available earthworm *Dendrobaena veneta* using techniques such as light, fluorescence and electron microscopy along with both a chemically fixed tissue and life cell imaging. This paper is devoted to the analysis of F-actin and the microtubular cytoskeleton in the cysts that develop in the seminal vesicles. Additionally, experiments using cytoskeletal drugs such as colchicine, nocodazole, cytochalasin D and latrunculin A showed that the general architecture of cysts is not altered after drug treatment. The only detectable effects were the disorganization of the microtubular manchette and the presence of late spermatid nuclei within the cytophore.

## Materials and methods

### Animal material

Commercially obtained, adult (marked as size no. 4) specimens of the earthworm *Dendrobaena veneta*, Rosa 1839 were used. The earthworms were kept in their original boxes in laboratory conditions for several days/weeks.

### Light and electron microscopy

Dissected seminal vesicles were fixed in 2.5 % glutaraldehyde in a 0.1 M phosphate buffer (pH = 7.4) for 24 h at room temperature. After fixation, the material was rinsed several times with a mixture of 50 ml 0.1 M phosphate buffer and 50 ml of ddH_2_O to which 4.6 g saccharose was added. Afterwards, the material was postfixed for 2 h in a mixture of 1 % OsO_4_ in a 0.1 M phosphate buffer, dehydrated in a graded series of ethanol replaced by acetone and then embedded in an Epoxy Embedding Medium Kit (Sigma, St. Louis, MO, USA). Semi-thin sections (1 μm thick) were stained with methylene blue and then examined under an Olympus BX60 microscope equipped with an XC50 digital camera and CellSense Standard software. After contrasting with uranyl acetate (15 min) and lead citrate (20 min), ultra-thin sections (70 nm thick) were examined using a Hitachi H500 transmission electron microscope.

### Visualization of microtubules on Steedman wax sections

The dissected seminal vesicles were fixed in 4 % formaldehyde, which was freshly prepared from paraformaldehyde, in PBS for 30 min at room temperature for all the cytoskeletal staining.

Fixed seminal vesicles were dehydrated in a graded series of ethanol: 30, 50, 70, 90 and 96 % for 15 min each and then 2× 1 h in 100 % ethanol. After that, the tissue was saturated in Steedman wax/ethanol solutions: 1:3 for 24 h, 1:1 for 24 h and 3:1 for another 24 h. For the final saturation, 100 % wax was used for 24 h. The seminal vesicles were then embedded in Steedman wax, left for polymerization and cut into 6- to 12-μm-thick sections on a microtome Zeiss HYRAX M40.

Before staining, the sections were mounted onto microscope slides and de-waxed by using a reverse series of ethanol: 100 % for 2× 15 min and 90, 70 and 50 %, ddH_2_O for 15 min each. The de-waxed sections were then washed with 1 % Triton X-100 in TBS (Tris-buffered saline) for 20 min and washed with pure TBS for 5 min and afterwards incubated for 1 h in 1 % BSA (Bovine Serum Albumin) in TBS.

Mouse anti β-tubulin antibody (T-4020; Sigma-Aldrich) was used to visualize the microtubules. Primary antibodies were diluted in 1 % BSA in TBS at a ratio of 1:100, applied onto slides and incubated for 24 h at 4 °C. After washing in TBS (5 min), the anti-mouse secondary antibodies, which were conjugated with Alexa Fluor 488 (Sigma-Aldrich) that was diluted in 1 % BSA in TBS at a ratio of 1:50, were applied (1 h at room temperature). Finally, after staining with antibodies, the samples were additionally stained with DAPI (1 μg/ml; Sigma-Aldrich) for 30 min in the dark.

### Visualization of F-actin and microtubules in the cyst suspension

After fixation (as described above), the seminal vesicles were torn manually with tweezers under a stereomicroscope in order to obtain the cyst suspension in PBS. The suspension was stained with a rhodamine-conjugated phalloidin (2 μg/ml; Sigma-Aldrich) for 45 min, washed with PBS (5 min) and additionally stained with DAPI (1 μg/ml) for 30 min. To detect microtubules, the same protocol and anti-tubulin antibody was used as described above. The germ-line cysts were applied onto microscopic slides using a cytocentrifuge MPW 223c (MPW MED. INSTRUMENTS, Poland) and were examined under an Olympus BX60 fluorescence microscope or under an Olympus FV1000 confocal microscope with a ×60/NA 1.30 sil objective. Z-stack images were generated using a 405-nm laser for DAPI, a 488-nm laser for Alexa Fluor 488 dye and 568-nm for rhodamine-conjugated phalloidin dye. 3D datasets were analyzed as volume-rendered datasets using Imaris (custom software developed by Bitplane Scientific Software, Zurich, Switzerland) and Fiji (freeware software).

### Staining of live cells

Before sectioning, the animals were narcotized in 50 % ethanol for 5 min. TubulinTracker Green and Hoechst 33342 (Life Technologies) were used to visualize tubulins and cell nuclei in living cells. After dissection, the seminal vesicles were quickly transferred to a cell culture medium (Dulbecco’s Phosphate Buffered Saline, DPBS; Sigma-Aldrich). The seminal vesicles were then torn manually with tweezers under a stereomicroscope to obtain the cyst suspension in DPBS. Both of the above-mentioned live cell dyes were diluted in DPBS at a ratio of 1:1000 and the staining time oscillated between 10 and 20 min. After staining, the cyst suspension was immediately applied onto microscopic slides using a glass pipette. The microscopic slides were examined under an Olympus BX60 fluorescence microscope or an Olympus FV1000 confocal microscope with a ×60/NA 1.30 sil objective. Z-stack images were generated using a 405-nm laser for Hoechst 33342 and a 488-nm laser for TubulinTracker Green.

### Experiments using cytoskeletal drugs

In order to examine the role of the cytoskeleton during the development of male germ-cell cysts, a series of experiments were performed. After the earthworms were narcotized in 50 % ethanol for 5 min and, after they were dissected, the freshly obtained seminal vesicles were incubated in DPBS to which cytoskeletal drugs had been added. Nocodazole and colchicine (Sigma-Aldrich) were used to disrupt the microtubule cytoskeleton. Cytochalasin D and latrunculin A (Sigma-Aldrich) were used to destabilize the microfilaments. According to the manufacturer’s protocol, the nocodazole and cytochalasin D and latrunculin A were initially dissolved in DMSO (dimethyl sulfoxide) and the colchicine in ddH_2_O. Then, all the drugs were dissolved in DPBS in order to obtain the working solutions: for nocodazole 20 μM and 100 μM; for colchicine 250 μM; for cytochalasin D 4 μM and 8 μM; and for latrunculin A 0.25 μM and 0.5 μM. For colchicine and nocodazole, the seminal vesicles were incubated for 6, 12, 24 and 48 h. and for cytochalasin D and latrunculin A for 6 h. Additionally, blind and control samples were prepared for each experiment. For the blind samples, the seminal vesicles were fixed (as described below) immediately after dissection without any incubation. For the standard control experiments, the seminal vesicles were incubated in a cell culture medium to which a cytoskeleton drug solvent was added only (DMSO and ddH_2_O, respectively) without the addition of any cytoskeleton drug. After all the experiments, the seminal vesicles were fixed in 2.5 % glutaraldehyde for light and electron microscopy or in 4 % formaldehyde, which was freshly prepared from paraformaldehyde, for immunofluorescence and cell suspension labeling. The experimental probes were then stained and analyzed as described above.

## Results

### General cyst organization

The process of spermatogenesis in clitellate annelids has been intensively studied at the ultrastructural level in recent decades and detailed summaries have been provided by Jamieson (1981, 1992, 2006) and Ferraguti (2000). In this paper, we only describe the general aspects of spermatogenesis in *D. veneta* with the emphasis on the structure of the cysts and the cytoskeleton organization. The terminology was adopted after Jamieson (1981) with a few modifications such as using cysts instead of “morulae” and intercellular bridges (IBs) instead of “zonulae collaris”.

**Fig. 1:**
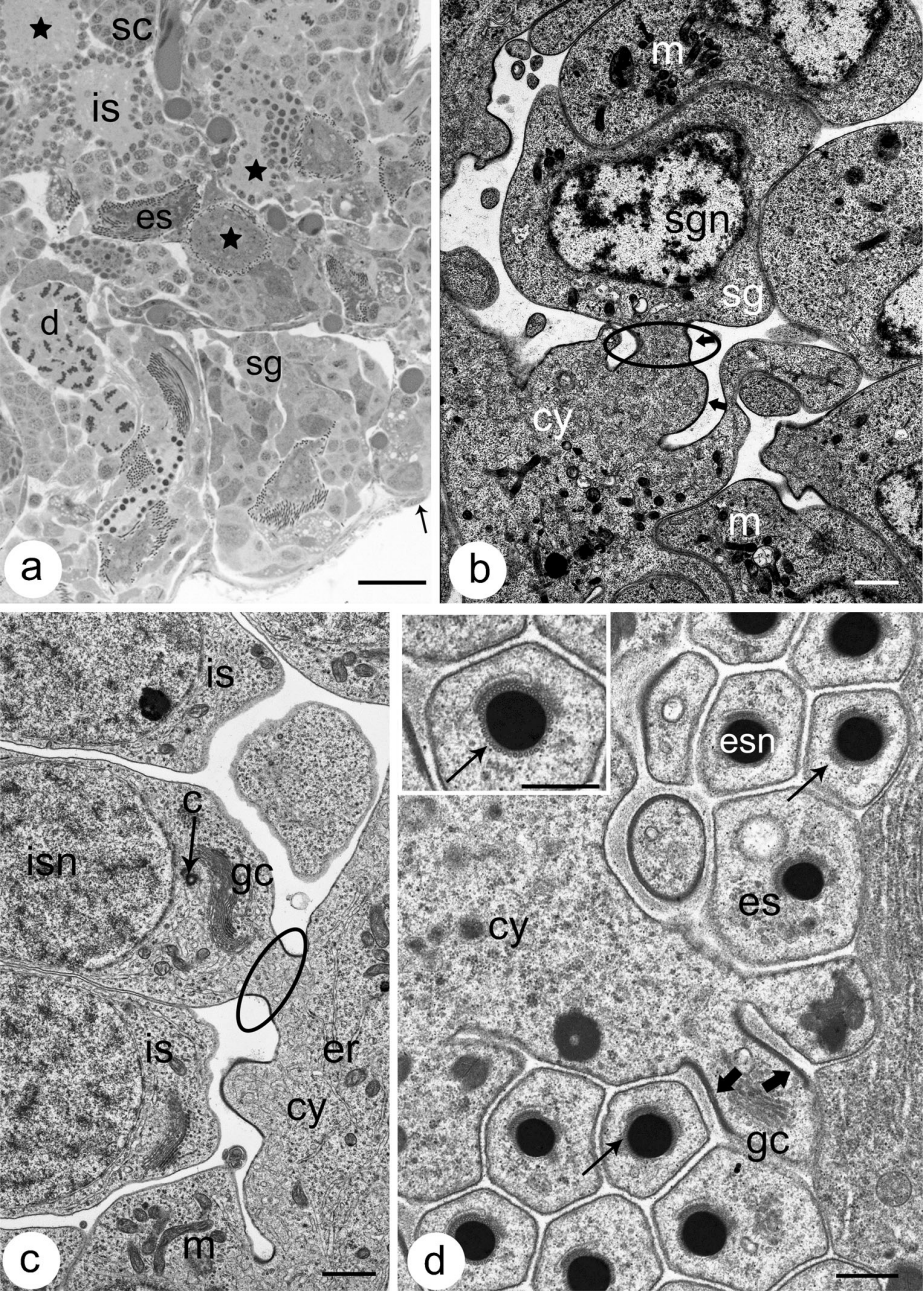
**a** A semi-thin section through the seminal vesicle. Vesicles are filled with hundreds of germ cysts. Germ cysts are at different stages of spermatogenesis and therefore spermatogonial cysts (*sg*), spermatocytic cysts (*sc*) and cysts with isodiametric (*is*) and elongate spermatids (*es*) can be observed. The center of a cyst is always occupied by a more or less voluminous anuclear cytophore (*stars*). *Arrow* marks the vesicle wall that is made up of somatic cells.* d* cyst with dividing cells. Light microscopy, methylene blue staining,* scale bar * 20 μm. **b** Ultrastructural detail of a spermatogonial cyst.* cy* cytophore,* m* mitochondria,* sg* spermatogonia,* sgn* spermatogonia nuclei. *Ellipse* the intercellular bridge; *arrows* the electron-dense bridge rim. Transmission electron microscopy (TEM),* scale bar* 1 μm. **c** A fragment of cysts with young (isodiametric) spermatids. *Ellipse* the intercellular bridge connecting the spermatid (*is*) with the cytophore (*cy*);* c* centriole,* er* endoplasmic reticulum,* gc* Golgi complex,* m* mitochondria,* isn* spermatid nuclei. TEM,* scale bar* 1 μm. **d** A portion of cysts with elongate spermatids (*es*) cross-sectioned at the level of their nuclei. Chromatin in elongate spermatids is tightly condensed and the nuclei (*esn*) are surrounded by a microtubular manchette (*thin arrows*). *Thick arrows* the electron-dense rim of the intercellular bridge;* cy* cytophore,* gc* Golgi complex.* Inset* higher magnification of nuclei enveloped by the manchette (*thin arrow*). TEM,* scale bar* 0.5 μm,* inset scale bar* 0.5 μm

**Fig. 2:**
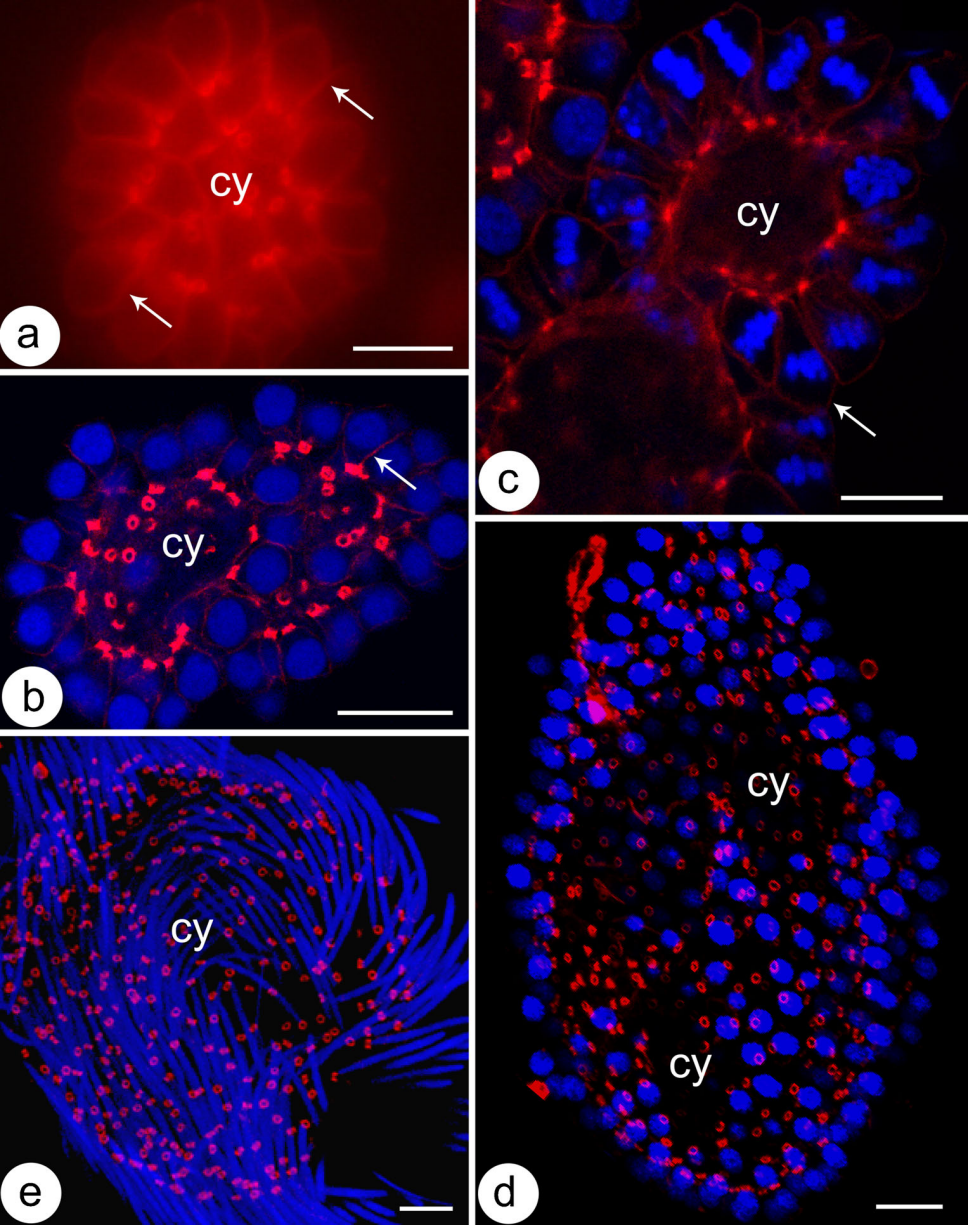
The germ-line cysts stained with rhodamine-conjugated phalloidin (**a**–**d**) and DAPI (**b**–**d**) in the consecutive stages of spermatogenesis*. Blue* cell nuclei or metaphase plates (in **c**), *red circles* intercellular bridges, thin arrows cortical F-actin,* cy* cytophore. **a** spermatogonial cyst, **b** spermatocytic cyst, **c** cyst with synchronously dividing cells, **d** cyst with isodiametric spermatids, **e** cyst with elongate spermatids. Single slices from confocal microscopy,* scale bar* 10 μm

**Fig. 3:**
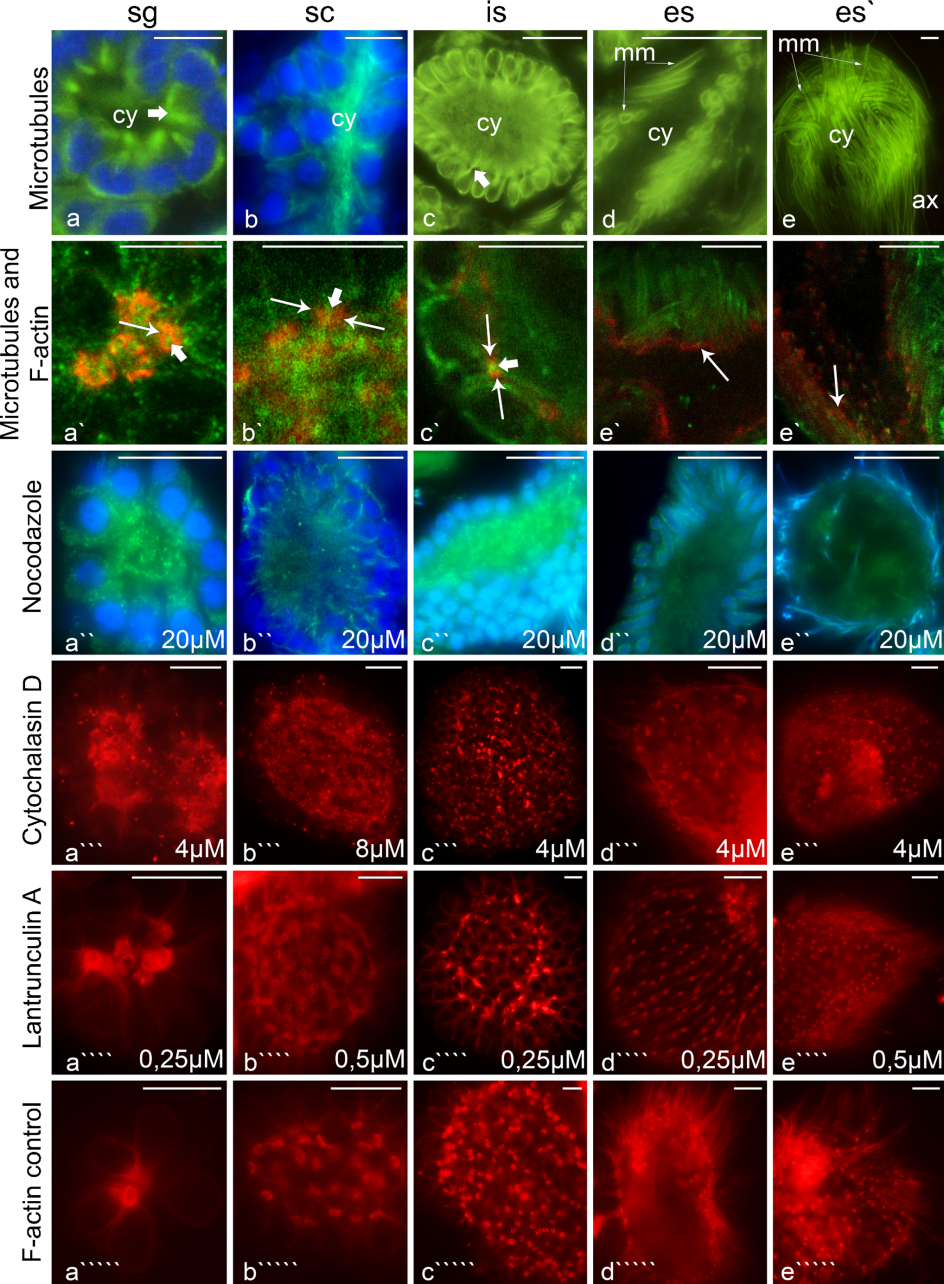
The distribution of microtubules and F-actin within the germ-line cysts not incubated (blind samples) and treated with the cytoskeletal drugs (incubation time = 6 h., concentrations are marked on the panels). Note that the general cyst morphology is the same in both cases. **a** spermatogonial cysts (*sg*), **b** spermatocytic cysts (*sc*), **c** cysts with isodiametric spermatids (is), **d** cysts with early elongate spermatids (*es*), **e** cysts with late elongate spermatids (*es’*); *blue* cell nuclei visualized by DAPI staining (**a**, **b**,** a''**–**e''**), *green* microtubules visualized by TubulinTracker Green (**e**) or by antibody against β-tubulin (**a**–**d**,** a'**–**e'**,** a''**–**e''**), *red* F-actin stained with rhodamine-phalloidin (**a'**–**e'**,** a'''**–**e'''**,** a''''**–**e''''**,** a'''''**–**e'''''**);* ax* axoneme,* cy* cytophore,* mm *microtubular manchette. *Thick arrows* microtubule concentrations in regions that correspond to the intercellular bridges, *thin arrows* intercellular bridges. **a**–**d** Steedman wax sections, the rest of the panels, whole mount preparations. Confocal (**a'**–**e'**) and fluorescence microscopy (the rest of the panels).* Scale bars* 10 μm

**Fig. 4:**
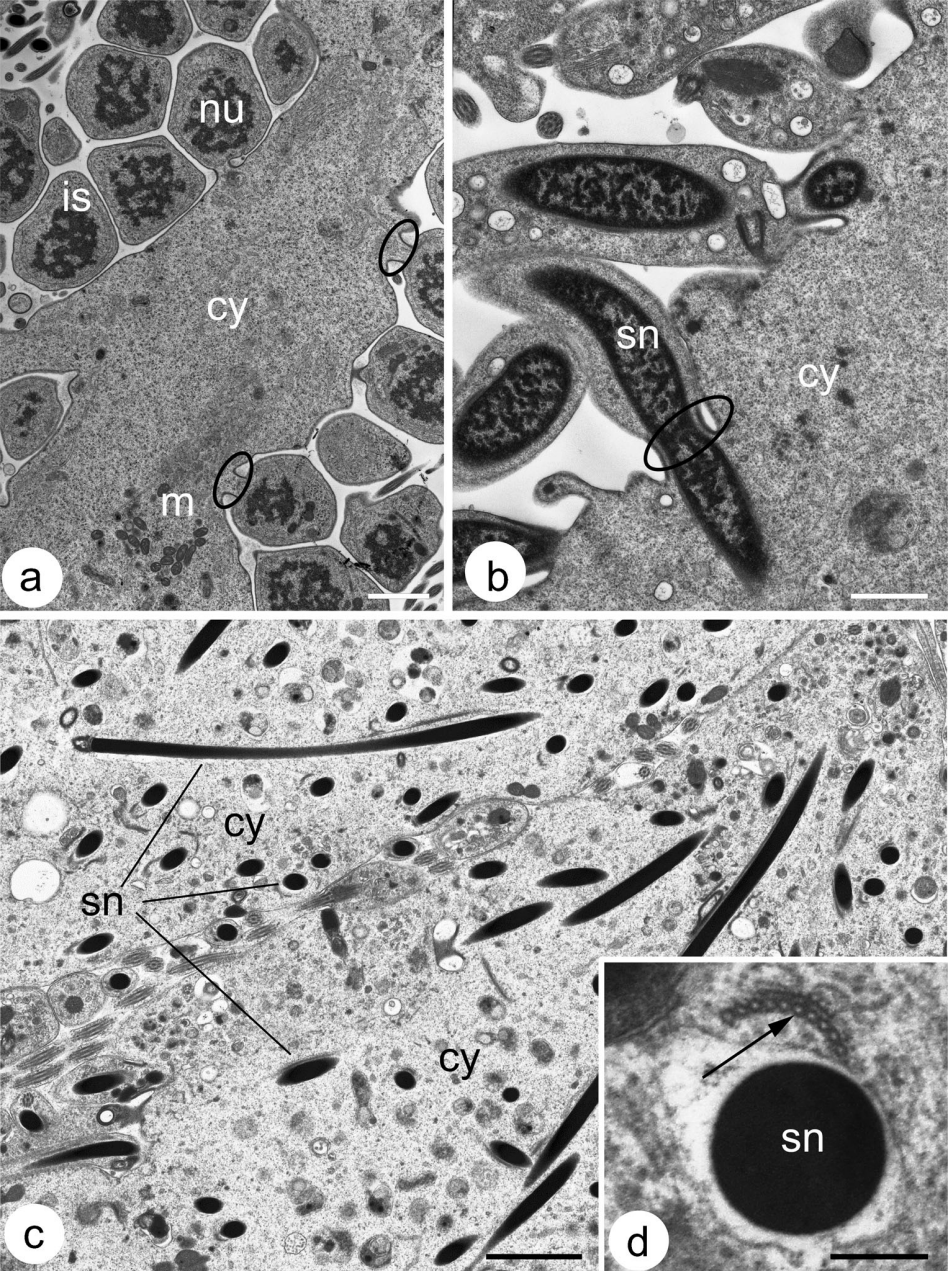
Ultrastructure of cysts incubated with nocodazole. **a**) The ultrastructure of a cyst incubated for 6 h (nocodazole concentration 100 μM). The cyst morphology is not altered;* is* isodiametric spermatids,* cy* cytophore,* m* mitochondria,* nu* cell nuclei. *Ellipses* the intercellular bridges.* Scale bar* 2 μm. **b** A fragment of a cyst with elongate spermatids that was incubated for 24 h (20 μM). The nuclei of spermatids (*sn*) inside the intercellular bridges (*ellipse*); the cytophore (*cy*) can be observed.* Scale bar * 1 μm. **c**) Fragments of two cysts with late elongate spermatids incubated for 48 h (20 μM). Numerous nuclei of the spermatids (sn) can be seen within the cytophore (*cy*).* Scale bar*  2 μm. **d** A cross-sectioned spermatid nucleus (*sn*) in the cytophore; note the remnants of the microtubular manchette (*thin arrow*) in close proximity of the nucleus.* Scale bar* 0.5 μm

**Fig. 5:**
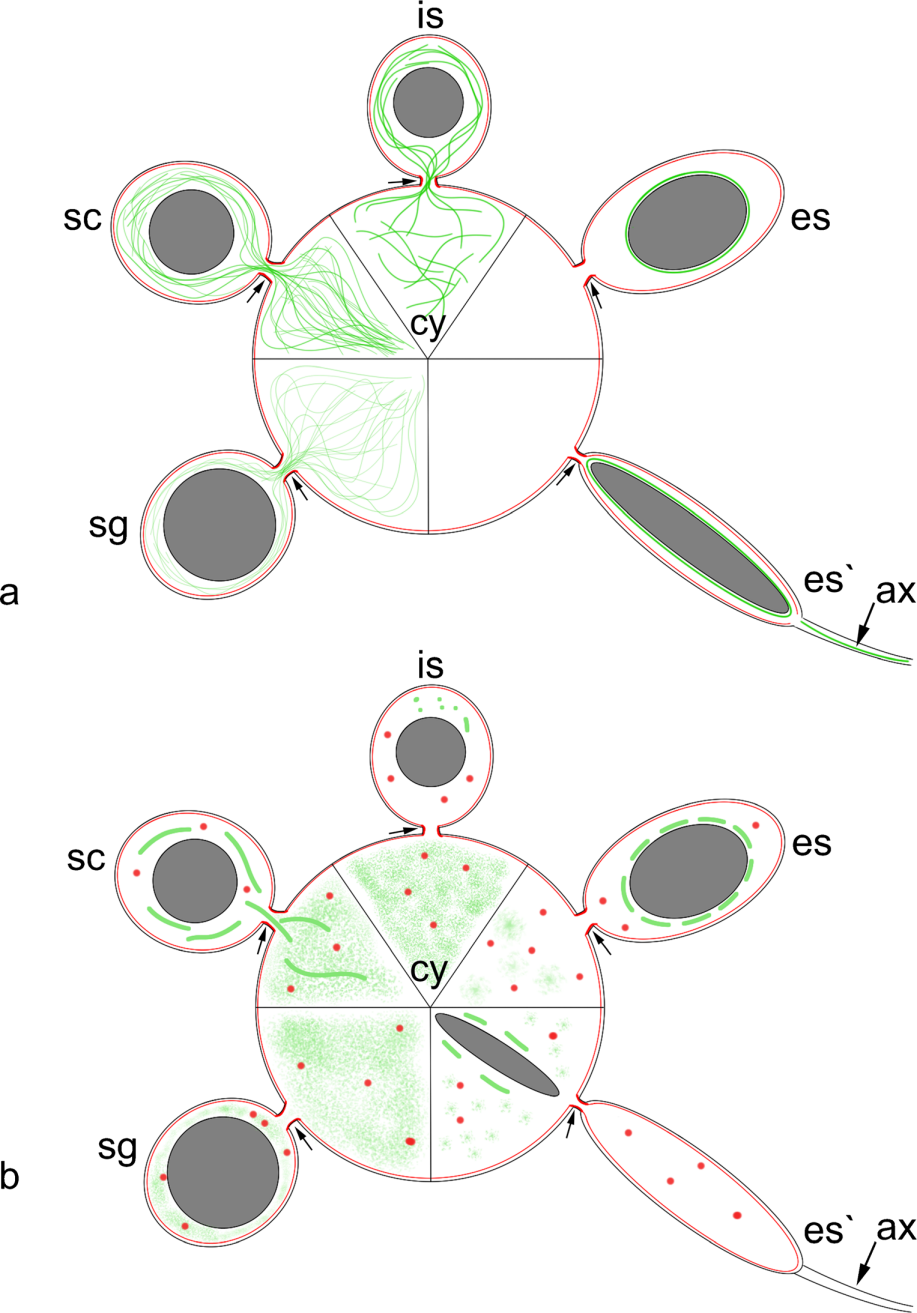
**a** The scheme of F-actin (*red*) and microtubule (*green*) distribution in the consecutive stages of cyst development. All cell organelles except for the germ cell nuclei (*grey*) and axoneme (*ax*) are omitted for clarity; cy cytophore, intercellular bridges are marked by arrows (for more details, see text); sg spermatogonial cyst, sc spermatocytic cyst, *is* cyst with isodiametric spermatids,* es* cyst with early elongate spermatids, *es′* cyst with late elongate spermatids. **b** Microtubule (*green*) and F-actin (*red*) distribution in cysts that were incubated in nocodazole and cytochalasin D, respectively. All cell organelles except for the germ cell nuclei (*grey*) and axoneme (ax) are omitted for clarity; cy cytophore, intercellular bridges are marked by *arrows* (for more details, see text);* sg* spermatogonial cyst,* sc* spermatocytic cyst,* is* cyst with isodiametric spermatids,* es* cyst with early elongate spermatids, *es′* cyst with late elongate spermatids

The seminal vesicles of *Dendrobaena veneta* are roughly oval structures that are located in segments IX–XI. We usually found three pairs of vesicles per specimen; however, in some cases, there were four pairs of vesicles or a number of vesicles were unpaired. There were hundreds of germ-line cysts within the vesicles (Fig. 1a). All the germ cells that were clustered in a single cyst were at the same phase of spermatogenesis; however, there was no synchrony between the cysts, i.e., cysts with a different number of clustered cells (i.e., cells in different phases of spermatogenesis) were observed in the same vesicle (Fig. 1a). Generally, in all the germ-line cysts that were observed, the center was occupied by an anuclear cytophore (Figs. 1, 2, 3, 4, 5). The shape and dimensions of the cytophore changed during the successive stages of spermatogenesis (see below). The gem cells were clustered around the cytophore and, as a rule, each germ cell was connected to the cytophore by one IB (Figs. 1, 2, 3, 4, 5).

The first step of study was to identify the cysts that were in the consecutive stages of spermatogenesis and to determine the cell number in the cysts. In order to find out how many cells constituted each spermatogenic stage, we counted the nuclei in 10 DAPI-stained cysts that contained elongate spermatids (these cysts are the easiest to identify). The number of cell nuclei in the elongate spermatid cysts varied between 248 and 255 (Table 1). Taking into account the synchronic divisions of germ cells that were observed within a given cyst (Figs. 1, 2c), it may be assumed that the total number of cell divisions during spermatogenesis is eight, which would potentially produce 256 spermatids. As a result, cysts that contained 64 cells were composed of primary spermatocytes, whereas secondary spermatocytes were clustered in the 128 cell cysts. Additionally, spermatogonial cysts were also observed within the seminal vesicles; these were 8-, 16- and 32-celled cysts (Fig. 1a, b). Cysts that had fewer than eight cells were never observed in the seminal vesicles.

**Table 1:** The number of cells (cell nuclei) that were counted in the ten cysts that contained elongate spermatids; the cysts were stained with DAPI for counting and analyzed using confocal microscopy

Cyst	Number of cells
1	250
2	254
3	249
4	252
5	250
6	255
7	248
8	250
9	249
10	252

The spermatogonia had large nuclei with dense chromatin that was embedded in a transparent matrix and their cytoplasm contained numerous small mitochondria and Golgi complexes, whereas cisternae of ER were rare (Fig. 1b). The cytophore in the spermatogonial cysts were small and had the form of an irregular mass of cytoplasm (Figs. 2a, 3a–a''''') that contained numerous ribosomes, mitochondria, short ER cisternae and lysosomes (Fig. 1b). The primary spermatocytes were easy to identify at the electron microscopy level where synaptonemal complexes were visible within their nuclei (not shown). However, at the light microscopy level, the clear distinction between primary and secondary spermatocytes was usually difficult and therefore we generally only identified the spermatocytic cysts (Figs. 1a, 2b, c, 3b–b'''''). At the light microscopy level, cysts that had isodiametric (young) and elongate (late) spermatids were the easiest to identify (Figs. 1a, 2d, e, 3c–c''''', d–d''''', e–e'''''). The isodiametric spermatids were roughly rounded cells that were connected to the cytophore, which was irregular or rounded in shape (Figs. 1a, c, 2d, 3c–c'''''). The nuclei of these spermatids were oval and the chromatin was not condensed. Rough ER cisternae and small mitochondria were scattered within the cytoplasm, while the Golgi complex and centrioles were located close to the proximal (anterior) end of the cell, i.e., the pole of the cell connected to the cytophore via the IB (Fig. 1c). Numerous mitochondria and cisternae of endoplasmic reticulum were observed within the cytophore of the isodiametric spermatids (Fig. 1c). The process of cell transformation into spermatozoon occurred intensively in the elongate spermatids (Figs. 1d, 2e, 3d–d''''', e–e''''') (see Jamieson 1981; Ferraguti 2000 for details). The following structures along their proximal–distal axis could be observed within the transforming spermatids: the developing acrosome, an elongated and condensing nucleus, mitochondria, the centriolar complex and the axoneme (not shown). The acrosome and nucleus in the elongate spermatids were enveloped by a microtubular manchette (Fig. 1d). The cytophore in these cysts reached its maximal dimensions (Fig. 2e). It was spherical and contained numerous mitochondria, ER cisternae and organelles that had a dense content and that were determined to be lysosomes (not shown). The IBs that connected the germ cells during all of the stages of spermatogenesis had a similar ultrastructure, namely, the cytoplasm strand was surrounded by an electron-dense layer of fibrous material that lined the plasma membrane, which is the so-called bridge rim (Fig. 1b–d). Cell organelles such as mitochondria, ER and Golgi complexes were observed within the bridges (Fig. 1b–d).

### F-actin and microtubule distribution

In order to visualize the F-actin, the cysts were labeled with rhodamine-coupled phalloidin (Figs. 2a–e, 3a'–e', a'''''–e'''''). The F-actin in all of the stages of cyst development studied was found in the cortical layer of germ cells and the cytophore (Figs. 2a–c, 3a'–e', a'''–e'''_,_ a''''–e'''', a'''''–e'''''). Additionally, a strong fluorescent signal in the form of rings came from the area where the germ cells were connected to the cytophore (Figs. 2a–e, 3a'–e', a'''–e'''_,_ a'''''–e''''', a'''''–e'''''). The F-actin-enriched rings corresponded to the electron-dense fibrous material that formed the IB rim (Fig. 1b). It should be stressed that we always observed just one F-actin ring corresponding to the IB between a given germ cell and the cytophore (Figs. 2, 3; see also Electronic Supplementary Material, Fig. S1). Multiple bridges between a given germ cell and the cytophore or between adjacent germ cells were never observed.

Two methods were used to detect the microtubules (MTs) – immunofluorescence using antibody against β-tubulin and live cell imaging using TubulinTracker Green. Both methods produced comparable results (Fig. 3a–e, a'–e'). The distribution of MTs changed during the consecutive stages of spermatogenesis. In the spermatogonial cysts, MTs were observed to be distributed within the germ cell cytoplasm and within the cytophore (Fig. 3a–a'). However, a high condensation of MTs was detected in the areas where the germ cells connected to the cytophore, i.e., within the IBs (Fig. 3a'; ESM, S1). On the other hand, in the dividing spermatogonial cysts, the MTs only formed mitotic spindles and no MTs were found in the bridges or within the cytophore (not shown). In the spermatocytic cysts, MTs were distributed both within the cytoplasm of the germ cells and within the cytophore, whereas no accumulations of MTs were observed in the areas that corresponded to the IBs (Fig. 3b–b'). In cysts that were composed of isodiametric spermatids, the MTs formed a dense network in the cortical cytoplasm of the germ cells and in the cytophore (Fig. 3c–c'). Specifically, prominent bundles of MTs passed from the cytoplasm of the isodiametric spermatids into the cytophore (Fig. 3c–c'). As was shown at the ultrastructural level, MTs form a manchette that tightly surrounds the transforming cell organelles such as acrosome and the nucleus in elongate spermatids (Fig. 1d); they also encompass the mitochondria and finally the MTs form the axoneme. Immunofluorescence and TubulinTracker Green labeling confirmed these observations (Fig. 3d–d', e–e'). MTs were not found in the cytophore and in the areas that corresponded to the IBs in either the late elongated spermatids or at the onset of the elongation of the spermatids (Fig. 3d–d', e–e').

Figure 5a presents the scheme that summarizes the changes in the F-actin and MTs distribution within germ-line cysts during the consecutive stages of spermatogenesis.

### Experiments using cytoskeletal drugs

The first step in conducting the experiments using cytoskeletal drugs was to elaborate the methodology of the germ-line cyst culture or outstay in vitro. We found that the germ-line cysts together with coelomic fluid (cyst suspension), which were freshly obtained from the seminal vesicles, mixed with DPBS and kept in small plastic vials, were vital for at least 48 h. The ultrastructure of spermatogonia, spermatocytes and spermatids, which were kept in vitro for 6, 12, 24 and 48 h, respectively, did not differ from the one that was found in the non-cultured cysts (not shown).

In order to disrupt the organization of MTs and microfilaments, seminal vesicles that had a developing germ-line cyst were incubated in media that contained nocodazole, colchicine, cytochalasin D or latrunculin A. The incubation times and the doses that were used varied from 6 to 48 h and from 5 to 250 μM, respectively (see “Materials and methods” for details). The use of colchicine produced no detectable results (not shown). The cyst architecture after treatment with cytochalasin D and latrunculin A also remained unaltered (Fig. 3a'''–e''', a''''–e'''', a'''''–e'''''); however, numerous granular rhodamine-phalloidin positive accumulations were found within the germ cells and the cytophore, especially after incubation in cytochalasin D (Fig. 3a'''–e'''). The general organization of the cysts that were incubated with nocodazole also did not change – the morphology of the cysts appeared to be unchanged (Fig. 4a). However, when it was visualized using immunofluorescence, the microtubular cytoskeleton was found to be severely damaged (Fig. 3a''–e''). In the spermatogonial and spermatocytic cysts and also in the cysts that were composed of isodiametric spermatids that were incubated in nocodazole, the detected tubulin was mainly globular in form and MTs were observed only occasionally (Fig. 3a''–c''). No bundles of MTs that passed the intercellular bridges were observed (Fig. 3a''–e''). MT manchettes were detected in the cysts that contained spermatids at the onset of their elongation that were treated with nocodazole; however, the immunofluorescence visualization suggested that the manchettes did not fully surround the spermatid organelles (Fig. 3d''). These results were confirmed by ultrastructural analysis (Fig. 4c, d). Manchettes that enveloped organelles were not detected in the cysts composed of elongate spermatids by the immunofluorescence methods (Fig. 3e''). A weak signal from tubulin was observed within the cytophore of these cysts only (Fig. 3e''). As was demonstrated by the ultrastructural analysis, these signals may have come from the manchettes that were partially destroyed and passed through IBs to the cytophore (Fig. 4d).

The ultrastructural observations and immunofluorescence methods revealed that, within the cysts with the elongate spermatids that were treated with nocodazole, the nuclei of the spermatids had a tendency to pass through the intercellular bridges into the cytophore (Fig. 4b). In such cysts, numerous nuclei of the spermatids were detected within the cytophore (Fig. 4c). In the blind and control experiments, no nuclei of the spermatids were ever observed within the cytophore (not shown). The distribution of tubulin in the cysts after nocodazole treatment is schematized in Fig. 5b.

## Discussion

The general and ultrastructural aspects of sperm formation that were found for *D. veneta* in this study are in accordance with the general description of spermatogenesis in clitellate annelids that was summarized by Jamieson (1981, 1992, 2006) and Ferraguti (2000). One aspect of spermatogenesis in *D. veneta* is especially noteworthy. Usually, there are 128 spermatids per cyst in Lumbricida and other clitellate annelids (Jamieson 1981). We found that there are 256 spermatids per cyst in the species studied here. This means that there is one extra mitotic division of spermatogonia in *D. veneta* in comparison with other earthworms such as *L. terrestris* (Walsh 1954; Krüger et al. 2008). Among Oligochaeta, 256 spermatids that were clustered in a cyst have also been reported in two lumbriculid species (Ferraguti 2000).

The organization and role of the cytoskeletal elements in developing male gametes have been intensively studied, especially in vertebrates (mainly mammals) and in model invertebrates such as *D. melanogaster* (recently reviewed in Lie et al. 2010; Sun et al. 2011; Sperry 2012; O’Donnell and O’Bryan 2014). Usually, these descriptions concentrate on the mitotic and meiotic divisions of the germ cells and on late spermatogenesis, i.e., spermiogenesis when the haploid spermatids undergo rapid changes in cell organization such as cell elongation, chromatin condensation, the formation of the acrosome and flagellum. It is well known that the cytoskeleton plays an active role in all these processes (Sperry 2012). It has also been demonstrated that the correct organization and regulation of the cytoskeleton dynamics is essential for male fertility (see Lie et al. 2010; O’Donnell and O’Bryan 2014 for details). To the best of our knowledge, the results presented here are the first comprehensive description of the F-actin and microtubular cytoskeleton in male germ-line cysts that have a cytophore.

### F-actin

Using rhodamine-coupled phalloidin, we found strong F-actin positive signals from the IBs in the form of ring-like structures. The presence of F-actin delimiting the IBs interconnecting germ cells appears to be a widespread phenomenon of gametogenesis, especially in oogenesis (Haglund et al. 2011). F-actin has been detected in the IBs in the male cysts of mammals such as rat, squirrel and mouse (Haglund et al. 2011), whereas F-actin is absent in fully formed bridges in fruit fly males (Hime et al. 1996; Eikenes et al. 2013). Using rhodamine-phalloidin staining, F-actin rings within the IBs have been found in clitellate annelids in both female (e.g., the fish leech *Piscicola geometra*, Spałek-Wołczyńska et al. 2008; the sludge worm *Tubifex tubifex*, Urbisz et al. 2015) and male cysts (the earthworm *Lumbricus terrestris* – Krüger et al. 2008; the sludge worm, Boi et al. 2001). F-actin rings appear to stabilize the bridges and keep them permeable, which is, in turn, a prerequisite to the efficient transfer of cytoplasm between germ cells. While the presence of F-actin in the cortical cytoplasm and in the IBs in cysts that are composed of spermatogonia, spermatocytes and spermatids is not unusual, surprisingly, we were unable to detect F-actin by rhodamine-phalloidin staining in any other localizations within the cysts that clustered around the spermatids. Krüger et al. (2008) analyzed the distribution of the gelsolin-related protein (EWAM-P1) and both G- and F-actin using rhodamine-phalloidin and immunofluorescence in the developing male cysts of *L. terrestris*. Like our studies, they found actin in the IBs but they also found actin in the proximal and distal parts of the spermatocytes, in the cytophore of spermatid cysts and in the distal part of the spermatids. Additionally, the pattern in which the anti-actin antibodies marked the periphery of the cell nucleus depended on the stage of spermatogenesis (Krüger et al. 2008). According to the authors, the actin concentrations in the distal poles of spermatocytes and spermatids may be connected with the activity of the Golgi apparatus and the actin that is localized in close proximity to the spermatid nuclei seems to “flow” from the spermatids towards the cytophore (Krüger et al. 2008). Additional studies using the anti-actin antibodies should clarify whether the localization of G- and F-actin in the developing male cysts of *D. veneta* is similar to that found in *L. terrestris* or whether the actin distribution is species-specific. Generally, it may be stated that the actin cytoskeleton in the male cysts of earthworms is not especially complex; no actin-rich structures such as, e.g., acroplaxome, actin cones or acroframosome, which are known from developing sperm of the other animals (see Sun et al. 2011 for a review), have been found. It is worth mentioning that a complex F-actin cytoskeleton has been found in female germ-line cysts that have a central cytoplasmic core in both annelids (*T. tubifex*; Urbisz et al. 2015) and nematodes (*C. elegans*; Wolke et al. 2007).

### Microtubules

The dynamics of MTs and MAPs (microtubule-associated proteins) are indispensable for the correct sperm formation; the role of MTs and MAPs in Sertoli cells, during germ cell divisions, in nuclear elongation, cytoplasmic redistribution and flagellar formation in spermatids are well known, especially in rodents (see Lie et al. 2010; Sperry 2012; O’Donnell and O’Bryan 2014 for details). Much less is known about the dynamics of MTs in invertebrate spermatogenesis, in which MTs are primarily recognized at the ultrastructural level and special attention is usually focused on the manchette, which is a transient microtubule-rich structure that tightly envelops the elongating spermatid nucleus. Our studies showed that MTs not only form the manchette in elongating spermatids but also reorganize dynamically during the subsequent stages of spermatogenesis. In spermatogenic cysts, MTs form a dense network in the germ cells and within the cytophore, in which they are highly concentrated in the areas that correspond to the IBs. During the meiotic phase, the network of MTs is also well developed in both the germ cells and the cytophore; however, no specific concentrations of MTs were found in the bridges. At the onset of spermiogenesis, the prominent microtubular bundles pass through the IBs and radiate into the cytoplasm of the cytophore (Fig. 5a). Later in spermiogenesis, MTs were only found in the manchette and the axoneme; no MTs were detected in the bridges or in the cytophore (Fig. 5a). Such a distribution of MTs is certainly connected with the transfer of cytoplasm and the formation and functioning of the cytophore. It is widely accepted that the cytophore is formed due to an accumulation of the cytoplasm of germ cells in the cyst center (Jamieson 1981; Ferraguti 2000; Świątek et al. 2009). The cytophore enlarges in the subsequent stages of spermatogenesis, e.g., the volume of the cytophore that interconnects the secondary spermatocytes increased at least 22-fold in *E. foetida* in comparison with spermatogonial cysts (Martinucci et al. 1977). The presented studies, together with the ultrastructural observation of MTs within the intercellular bridges that interconnect early spermatogonia in the testis of *E. foetida* (Martinucci and Felluga 1975), suggest that the microtubular cytoskeleton is responsible for the transfer of organelles and macromolecules. It should be stressed that the transfer between germ cells and cytophore is most probably bidirectional. The cytoplasm with the cell organelles, e.g., mitochondria, flows from the developing germ cells towards the cytophore. On the other hand, it has been suggested that some macromolecules such as proteins (e.g., tubulins) or glycogen are produced in the cytophore and then transferred to the germ cells (Martinucci et al. 1977). The presented observations, which show that MTs were not detectable during late spermiogenesis either within the IBs or the cytophore, suggest that there is no transfer (or that the transfer is weak and/or not MTs dependent) between the elongated spermatids and the cytophore and vice versa. At this time, the spermatozoa are almost fully formed and are ready to detach from the cytophore (Martinucci et al. 1977; own unpublished results).

### Drug experiments

In order to study the role of microfilaments and MTs, we incubated the germ-line cysts in media containing cytoskeletal drugs such as cytochalasin D and latrunculin A, which affect the microfilaments and colchicine and nocodazole, both of which affect the dynamics of the MTs (see Lie et al. 2010 for literature and details). Surprisingly, although we used several different working concentrations and the time of incubation was as long as 48 h (the time was limited due to the vitality of the cysts in an artificial media), none of the drugs that were used altered the general geometry of the cysts (Fig. 5b). The cyst morphology in the blind samples (without any incubation) under the control conditions (cysts incubated without drugs) and in the experimental probes was very similar; the shape of both the germ cells and the cytophore was still stage-specific and not altered. Weak cytochalasin D and latrunculin A incubation effects were found within the germ cells and the cytophore in the form of numerous granular rhodamine-phalloidin-positive accumulations. More prominent effects were found after the incubation of the cysts in nocodazole: in this case, numerous spermatid nuclei were found within the cytophore in late spermiogenesis. It is known that the efficacy of cytoskeletal drugs, especially those that alter MTs, is organism-specific, i.e., in some organisms the drugs alter the cytoskeleton organization whereas other organisms remain resistant (Dostál and Libusová 2014). The mechanisms of resistance are especially well known in some cancer lines (Pellegrini and Budman 2005). Why cytochalasin D, latrunculin A and colchicine did not affect the cytoskeleton in *D. veneta* germ-line cysts remains unresolved. On the one hand, it is known that the MTs in spermatids are modified by acylation, polyglutamylation or detyrosination and are more resistant to depolymerization (see Sperry 2012 for details). On the other hand, the effects of colchicine treatment on spermiogenesis have been described in, e.g., molluscs via the disruption of chromatin condensation (Maxwell 1983), whereas the use of cytochalasin D in some flatworms (Stitt et al. 1991) and rodents (Russell et al. 1987, 1991; Ventelä et al. 2003) caused the formation of multinucleate spermatozoa or significantly inhibited the granule movement between neighboring cells. The next explanation is connected with the timing of the germ cell divisions. We do not know whether germ cells divide at least once during the maximal treatment period (48 h). If the cells do not divide, this might explain the absence of some drug effects. Further analyses with an extended period of incubation and using drugs that have different mechanisms of cytoskeleton disruption than those that were used in the presented study such as, e.g., colcemid or taxol, should explain whether the F-actin and MT in the male germ-line cysts of *D. veneta* are especially resistant to cytoskeletal drugs.

Using immunofluorescence, we showed that nocodazole caused the depolymerization of MTs; however, the cyst morphology was untouched, which shows that the MTs are not responsible for keeping the shape of the germ cells and the cytophore. Interestingly, while the nuclei of spermatogonia and spermatocytes did not change their positions, the nuclei of the elongated spermatids had a tendency to move away from the cells towards the cytophore in the nocodazole-incubated cysts. This suggests that not only the IBs prevent the spermatid nuclei from passing from the cells into the cytophore as was suggested based only on the ultrastructural observations (Martinucci et al. 1977) but also that the microtubular cytoskeleton is engaged in keeping them in a place. Most likely a manchette, a structure surrounding the spermatid nuclei in a diverse group of animals that is rich in MTs (e.g., annelids, molluscs, mammals; see Maxwell 1983; Ferraguti 2000; O’Donnell and O’Bryan 2014), is responsible for the positioning of the spermatid nuclei during late spermiogenesis. The structure and functions of the manchettes are best known in rodents, in which the manchette contains both MTs and F-actin and is engaged in, e.g., shaping the sperm head (Russell et al. 1991) and cytoplasmic redistribution (Kierszenbaum 2002). The microtubular manchette is also well developed in clitellate annelids (Jamieson 1981, 1992; Ferraguti 2000) and some ultrastructural observations suggest that there is a relationship between the MTs, chromatin condensation and nuclear morphogenesis (Ferraguti and Lanzavecchia 1971). Our experiments showed that nocodazole disrupts the integrity of the manchette and that some fragments of the manchette, together with spermatid nuclei, also move from the spermatids into the cytophore. It may be supposed that, in natural conditions, the manchette prevents the spermatid nuclei from moving while the residual cytoplasm is transferred towards the cytophore; when the manchette is disrupted, it flows (in an actin-dependent manner?) into the cytophore together with the nucleus. A functionally similar mechanism that prevents the germ cell nuclei from moving towards the IBs during mass cytoplasm transfer is known from the ovaries of some insects; e.g., in some hymenopterans, MTs form cages around the nurse cell nuclei and after colchicine treatment, the nuclei move towards the IBs (for details, see Adamska and Biliński 1997; Biliński and Jaglarz 1999). Finally, it should be added that there are some ultrastructural reports that show that, in natural conditions, the nuclei of the spermatids in some clitellate annelids may extend through the intercellular bridges into the cytophore (e.g., early spermatids in the earthworm *Microchaetus pentheri*, Hodgson and Jamieson 1992 and in *E. foetida*, Martinucci et al. 1977) or that even spermatid nuclei have been observed within the degenerating cytophore (Martinucci et al. 1977). The nuclei of the female germ cells have also occasionally been found within the cytophore in late oogenesis in several clitellate annelids (Świątek 2008; Urbisz and Świątek 2013; Urbisz et al. 2014). It has been suggested that such a phenomenon is connected with defective mechanisms of spermatid detachment that is related to the relaxation of an IB (Martinucci et al. 1977) or with the degeneration of germ cells and/or the disturbance of the cytoplasm transfer from the nurse cells to the oocytes via the cytophore (Urbisz and Świątek 2013).

## Conclusions

The results that were obtained show that:

1. F-actin is enriched in the cortical cytoplasm and forms distinct rings in the IB rims in all the stages of cyst development that were studied (Fig. 5a);
2. the distribution of the MTs changes dynamically in the consecutive stages of spermatogenesis (Fig. 5a);
3. cytoskeletal drugs such as colchicine, nocodazole, cytochalasin D and latrunculin A do not alter the general morphology of cysts;
4. after treatment of the germ-line cysts with nocodazole, spermatid nuclei and manchette fragments were found within the cytophore (Fig. 5b);
5. it appears that the MTs play the main role in cytoplasm/organelle transfer between the cells and the cytophore and prevent the nuclei of spermatids from passing through the IBs.

## Electronic supplementary material

Below is the link to the electronic supplementary material.

Fig. S1 An animation of serial optical slices from spermatogonial cysts collected by confocal microscopy. *Green* microtubules visualized by antibody against β-tubulin, *red* F-actin stained with rhodamine-phalloidin. (AVI 786 kb)
